# Nutritional considerations for designing ketogenic dietary interventions for people with Autosomal Dominant Polycystic Kidney Disease

**DOI:** 10.1007/s40620-025-02378-3

**Published:** 2025-08-10

**Authors:** Jessica Dawson, Anna Rangan, Gopi K. Rangan

**Affiliations:** 1 Trials Centre, NHMRC Clinical, University of Sydney, Sydney, Australia; 2 Dept of Nutrition and Dietetics, St George Hospital, Sydney, Australia; 3 Nutrition and Dietetics, Faculty of Medicine and Health, Charles Perkins Centre, The University of Sydney, Sydney, Australia; 4 Michael Stern Laboratory for Polycystic Kidney Disease, Centre for Transplant and Renal Research, Westmead Institute for Medical Research, The University of Sydney, Sydney, Australia

**Keywords:** Autosomal-dominant Polycystic Kidney Disease, Ketogenic diet, Nutrition

## Abstract

Autosomal Dominant Polycystic Kidney Disease (ADPKD) is a progressive genetic disease with few effective treatments available to slow the decline in kidney function. In ADPKD, there has been increasing interest in ketogenic diets, largely based on experimental data showing favorable effects on cyst growth and kidney function. High-quality clinical trials of sufficient duration using clinically relevant endpoints (estimated glomerular filtration rate [eGFR], kidney volume) are crucial to establish efficacy and safety in ADPKD. Although extensive reviews have been published about potential mechanisms of ketogenic diets to slow ADPKD progression, there is no guidance on how these diets should be designed to align with the unique clinical and nutritional needs of people with ADPKD. Potential safety and feasibility concerns for ketogenic diets (risk for nephrolithiasis and cardiovascular disease) should be evaluated in clinical trials to determine whether adherence to a complex diet can be sustained over years. Prior to embarking on a clinical trial, careful development of an ADPKD-appropriate ketogenic diet is required to mitigate against these risks. Thus, the aim of this narrative review is to provide a framework for the specific nutritional factors that should be considered when developing and designing a ketogenic dietary intervention in future clinical trials involving ADPKD patients.

**Graphical abstract:**

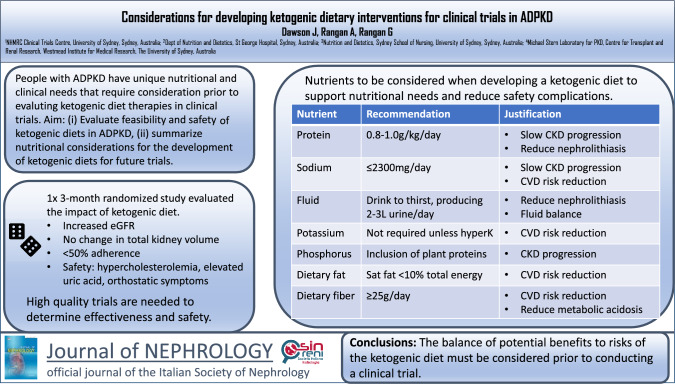

**Supplementary Information:**

The online version contains supplementary material available at 10.1007/s40620-025-02378-3.

## Introduction

Autosomal Dominant Polycystic Kidney Disease (ADPKD) is the most common inherited kidney disease, caused by pathogenic variants in *PKD1*, *PKD2,* or rarely other genes [1]. It accounts for ~ 10% of patients receiving chronic dialysis or kidney transplantation for kidney failure [1]. Pathogenic variants in one of the PKD genes lead to the formation and arginine vasopressin-dependent growth of fluid filled kidney cysts, causing progressive kidney enlargement and compressive chronic injury to the renal parenchyma [2]. Compared to other chronic kidney diseases (CKDs), estimated glomerular filtration rate (eGFR) declines faster in ADPKD, with most patients progressing to kidney failure at a median age of 58 years [2].

Dietary and lifestyle interventions to slow progression are highly prioritized by the ADPKD community [3, 4]. Specific recommendations include moderate protein (0.75–1.0 g/kg/day) and reduced sodium (< 2300 mg/day) intakes as well as maintaining adequate hydration to attenuate arginine vasopressin [5]. Table 1 summarizes evidence in ADPKD regarding protein and/or sodium restrictions [6–8] [9] Of these, only one randomized controlled trial (RCT), the Modification of Diet in Renal Disease (MDRD) evaluated the impact of diet (protein restriction) on disease progression [6]. Additionally, generic lifestyle modifications, including exercise and healthy eating patterns [Mediterranean diet or Dietary Approaches to Stop Hypertension (DASH)], are often recommended [10, 11], albeit with no clinical trial data in the ADPKD population.

**Table 1:** Randomized clinical trials using dietary interventions for slowing CKD progression or evaluating markers of the disease in individuals with ADPKD. Dietary RCTs evaluating interventions that slow disease progression

Dietary RCTs evaluating interventions that slow disease progression						
	Study design	Population	Intervention	Control	Primary Outcome	Secondary outcome
Klahr 1995*	RCT 26 months	Study A (n = 141) eGFR > 25-55 ml/min/1.73m^2^	LPD (0.58 g/kg/day)	Habitual protein diet (mean 1.3 g/kg/day)	Change in kidney function LPD: −5 ml/min/1.73m^2^ Control: −5.5 ml/min/1.73m^2^ (no difference) No association between protein intake and kidney function (+ 0.1 ml/min/yr;. p = 0.94)	
Klahr 1995*	RCT 26 months	Study B (n = 59) eGFR 13-24 ml/min/1.73m^2^	LPD (0.58 g/kg/day)	VLPD with keto-analogue supplements (0.28 g/kg/day)	Change in Kidney Function Low proteinLPD: −4.9 ml/min/1.73m^2^ Very low proteinVLPD: −4.0 ml/min/1.73m^2^ Between group diff p = 0.06 Marginal association of higher protein intake with kidney function decline (−3.1 ml/min/yr; p = 0.06)	% reaching kidney failure LPD: 69% VLPD: 60% (no difference)
Dietary RCTs evaluating urine and/or plasma markers of disease						
Amro 2016	RCT 2 weeks	n = 34 eGFR ≥ 60 ml/min/1.73m^2^	Low osmolar diet Sodium 1800 mg/day Protein 0.8 g/kg/day Low urea intake Personalized water prescription to produce mean urine osmolality of 280 mOsm/kg	Habitual diet	Copeptin Intervention: ↓ 6.2 pmol/L (SD 3.05) to 5.3 pmol/L (SD 2.7) (p = 0.02) Control: ↔ 4.7 pmol/L (SD 3.6) to 5.07 pmol/L (SD 4) (p = 0.2) Sig diff between arms at 2 weeks (p = 0.009)	Urine osmolality Intervention: sig ↓ from 426 mOsm/kg (SD 193) to 258 mOsm/kg (SD 117) (p = 0.01) Control: ↔ 329 mOsm/kg (SD 159) to 349 mOsm/kg (SD 139) (p = 0.3) Sig diff between 2 arms at 2 weeks (p = 0.007)
Geertsema 2024	Crossover RCT 10 weeks	N = 12 eGFR > 30 ml/min/1.73m^2^ and receiving Tolvaptan	Dietary intervention: - LPD (0.8 g/kg/day) - Low salt (< 6 g salt/day) Plus capsules containing - 6 g salt - 40 g protein 4 periods of dietary intervention plus: No capsules Salt capsule Protein capsule Salt and protein capsules	N/A	Urine volume Sig ↓ from baseline (5.9L, SD 1.2L) to: Low salt plus low protein 5.2L (SD 1.1L) (p = 0.004) Low salt 5.4L (SD 0.9L) (p = 0.04) ↔ Low protein −0.4L (p = 0.1)	Copeptin Sig ↓ from baseline (33.2 pmol/L, SD 10.2) Low salt plus LPD (−13.5 pmol/L, p < 0.001) Low salt (−10.4 pmol/L, p < 0.001) LPD (−5.7 pmol/L, p < 0.001) Measured GFR Sig ↓ from baseline (56 ml/min/1.73m2, SD 21) Low salt plus LPD (59 ml/min/1.73m2, SD 23; p = 0.01) ↔ mean arterial pressure ↔ quality of life Sig ↓ Urine osmolality from baseline (172 mOsm/kg, SD 41) Low salt plus LPD (126 mOsm/kg, SD 35; p < 0.001) Low salt (159 mOsm/kg, SD 36; p = 0.04) LPD (160 mOsm/kg, SD 31; p = 0.048) Sig Plasma osmolality from baseline (298 mOsm/kg, SD 8) Low salt plus LPD (−10 mOsm/kg, p < 0.001) LPD (−6 mOsm/kg, p = 0.01)

↓ Decrease;  ↔, no change; ↑ increase

*eGFR* estimated glomerular filtration rate, *LPD* low protein diet, *VLPD* very low protein diet

In recent years, there has been a surge of consumer interest in ketogenic diets in ADPKD. Nine narrative reviews [11–21] and one systematic review [22] have been published highlighting the potential mechanisms by which ketogenic diets could be a disease-specific intervention to reduce kidney cyst growth. To date, there are four clinical trials in ADPKD evaluating ketogenic or very low carbohydrate diets identified on the International Clinical Trials Registry (search conducted on 12 February, 2025). Whilst increasingly of interest, there has been no guidance on how to design these diets to align with existing dietary recommendations and clinical complications of ADPKD (particularly nephrolithiasis and cardiovascular disease). Therefore, prior to embarking on a clinical trial, it is crucial to identify which factors should be considered when designing a ketogenic diet that is appropriate for ADPKD.

The purpose of this narrative review is to provide guidance on designing a ketogenic diet to be used in clinical trials that is clinically and nutritionally appropriate for people living with ADPKD. This review will focus only on ketogenic dietary RCTs and will not discuss other related methods, such as caloric restriction, intermittent fasting or ketone supplements.

## Scientific rationale for a ketogenic diet in ADPKD

A ketogenic diet is very low in carbohydrates with varying amounts of protein and fat, with several versions being used in other conditions, such as epilepsy and weight loss [23]. Typically, the macronutrient composition of ketogenic diets is based on 60–90% of total calories being derived from dietary fat, ≤ 10% from carbohydrate (usually restricted to < 50 g of net carbohydrates per day), and 10–25% from protein (Table 2) [23–25]. In ADPKD, the aim of a ketogenic diet is to switch the primary energy source from carbohydrate to fat [15]. The increased oxidation of fatty acids and ketone body production, primarily β-hydroxybutyrate and acetoacetate, creates a state of ketosis [15]. In ADPKD, as kidney cystic cells are unable to sufficiently metabolize fatty acids, it has been hypothesized that a ketogenic diet could reduce the progression of kidney cyst growth and slow kidney function decline.[11, 17] In support of this hypothesis, in preclinical models of ADPKD a state of ketosis has led to cell death, loss of cystic fluid and reduced cystic kidney disease [11, 25]. Oral administration of β-hydroxybutyrate has also inhibited cyst growth in animals [26].

**Table 2:** Macronutrient composition of various diets

Diet	Fat	Carbohydrate	Protein
Standard dietary guidelines^70^	20–35%	45–64%	10–35%
ADPKD guidelines^5,10^	NR for total fat (< 10% from saturated fat)	NR	0.8–1.0 g/kg/day (equivalent to 10–12%)
CKD guidelines^5,31^	NR	NR	0.6–0.8 g/kg/day (equivalent to 8–10%)
Classic ketogenic^22^			
4:1	90%	2–4%	6–8%
*Modified ketogenic diets*^*22*^			
3:1	85–90%	2–5%	8–12%
2:1	80–85%	5–10%	10–15%
Modified Atkins Diet	60–65%	5–10%	25–35%
Low Glycemic Index Treatment	60–70%	20–30%	10–20%

The ketogenic ratio is defined as the ratio of grams of fat to grams of carbohydrate plus protein. 4:1 provides 4 g of fat for every 1 g of carbohydrate plus protein

% represent the proportion of calories of total energy intake

^*^As CKD protein guidelines vary this is based on an average protein intake of 0.6–0.8 g/kg/day and a total caloric requirement of 35 kcal/kg/day

*NR* no recommendation

## Published clinical trials involving ketogenic diets in ADPKD

A literature search was conducted using PubMed to identify relevant studies using a combination of Medical Subject Headings (MeSH) terms and keywords related to dietary interventions and ADPKD. Studies were limited to randomized controlled trials that employed a dietary intervention as the primary strategy. Currently there is no evidence to recommend the use of ketogenic diet to reduce the progression of ADPKD[22], with only two small short-term clinical trials and 1 retrospective observational study published (summarized in Table 3). The only RCT available (KETO-ADPKD) evaluated a 3-month ketogenic diet (n = 23) in ADPKD (mean 84 ± 24 ml/min/1.73 m^2^), and reported that ketogenic intervention reduced body weight (−7.2%) and improved eGFR (5.51% ± 11.4%) compared to the control diet (n = 21) or water fasting (n = 22) [27]. There was no difference in other clinical outcomes (total kidney volume [TKV], blood pressure, albumin-to-creatinine ratio). Adherence to the ketogenic diet was 47% at 3 months, determined by β-hydroxybutyrate levels being ≥ 0.8 mmol/L at all 3 study visits. The most common adverse side effects in the ketogenic diet group were “keto-flu” (symptoms associated with ketosis including headaches, nausea, irritability, and brain fog) (43%), orthostatic symptoms (17%), elevated uric acid levels (17%), and elevated cholesterol levels (including total cholesterol, LDL-C, VLDL-C, non-HDL-C) (17%) (Table 3).

**Table 3:** Main clinical trials of ketogenic diets in patients with APKD

	Study design	Population	Intervention	Comparator	Clinical Outcomes	Feasibility, Adherence	Safety and tolerance
Clinical trial							
KETO-ADPKD^26^	3-month 3-arm RCT	n = 66 51% male mean age 41.4yrs (9.5yrs) mean eGFR 84 ± 24 ml/min/1.73m^2^ mean htTKV 958 ± 651 ml/m	A) Ketogenic diet (< 5-7 g salt; 0.8 g protein; < 30 g carbohydrate; 3L water) B) 3-consecutive day water fast once per month, other days to eat ad libitum*,* < 5-7 g salt and 3L water	C) Control: Ad libitum diet with < 5-7 g salt, 3L water	↓ body weight in KD (−7.2%) vs control (+ 0.27%) (p = 0.007) Sig ↑ eGFR in KD (+ 5.51% ± 11.4%) vs control (−1.74% ± 11.7%) (p = 0.027) ↔ htTKV, BP, UACR	47% adherent (BHB levels ≥ 0.8 mmol/L at all 3 study visits) 61% adherent (BHB levels ≥ 0.5 mmol/L at all 3 study visits) Self-reported feasibility (score > 0 indicates feasible) 95% responses score > 0 in KD arm 85% responses score > 0 in water fast arm	Hypercholesterolemia: KD n = 4 ↑ Uric acid levels KD n = 4 Water n = 1 Control n = 1 Orthostatic-related symptoms KD n = 4 Keto-flu KD n = 10 Water n = 3 Symptomatic kidney stones KD n = 2 Appendicitis KD n = 1 Cyst infection Control n = 1
*Non-randomized intervention studies*							
Reset-PKD^71^	Pilot, non-randomized intervention study	n = 10 80% male Mean age 39.8 ± 11.6 yrs Mean eGFR 82.2 ± 23.5 ml/min/1.73m^2^ Mean TKV 2224 ± 1156 ml/m	14-day Ketogenic diet (fat: protein: carbohydrate – 10:4:1 (in grams), with 20–25 kcal/kg/day) Meals supplied Blueberries allowed if required for symptoms or BHB levels > 3.5 mmol/L Run in with “carbohydrate-rich” diet	3-day water fast with ad libitum water and 1 × low salt broth per day Meals/snacks supplied. Blueberries allowed if required for symptoms Run in with “carbohydrate-rich” diet	↓ body weight in KD and WF (p < 0.05) ↓ TLV in water fast arm (p = 0.016), no change in KD ↓ TLV/BW ratio in WF arm (p = 0.04) ↔ htTKV, liver cyst volume, blood pressure	↑ BHB levels pre and post HD (p = 0.009) but not WF (p = 0.06) Sig ↑ acetone pre and post KD diet (p = 0.02) and WF (p = 0.02) Feasibility 4/5 KD and 4/5 WF participants self-reported feasible 90% of patients reached the metabolic endpoint and/or the self-reported feasibility endpoint 80% of all patients reached both the metabolic endpoint and rated KDIs as feasible	↑ cholesterol (p = 0.015) and LDL-C (p = 0.028) in KD arm ↔ HDL-C, TG ↑ uric acid in KD (p = 0.028) and WF (P = 0.015) ↑ hunger in WF arm (p = 0.029)
Observational studies							
Strubl^69^	Retrospective, observational	n = 131 (n = 74 ketogenic diet and n = 52 time-restricted feeding) Median age 50 yrs (IQR 20) 30.5% male Median eGFR 57 ml/min/1.73m^2^ (IQR 32.5)	Self-reported experience with ketogenic type diets (ketogenic, time restricted feeding, caloric restriction) KD n = 74 TRF n = 52 CR n = 5	N/A	70 participants had pre and post eGFR measures with 45 (64%) having increased eGFR, 8 (11%) had no change and 17 (24%) had a decline in eGFR	80% report improvement in overall well-being 76% report implementation manageable 50% reported daily adherence, 42% skipped several times per month with 40% of breaks due to practical difficulties	Elevated cholesterol levels were the most common safety concern 67% report improvement in health issues, such as flank pain, fatigue 66% reported new health issues with uptake of diet, e.g., “keto-flu”. With 55% reporting these symptoms to be transient, 12% report symptoms persisted

*eGFR* estimated glomerular filtration rate, *htTKV* height adjusted total kidney volume, *TLV* total liver volume, *TLV/BW* total liver volume to body weight, *BHB* beta-hydroxybutyrate, *KD* ketogenic diet, *TRF* time restricted feeding, *CR* caloric restriction, *WF* water fasting, *HDL-C* high density lipoprotein cholesterol, *LDL-C* low density lipoprotein cholesterol, *TG* triglycerides

↓, reduction; ↑, increase; ↔, no change

## Considerations when designing ketogenic diets for clinical trials in ADPKD

High-quality RCTs are required to determine the safety and efficacy of ketogenic diets in ADPKD. In this regard, there are several important considerations when designing a ketogenic diet in a clinical trial in ADPKD. The ADPKD population have co-morbidities that are common to CKD (reduced eGFR, cardiovascular disease, dyslipidemia) combined with disease-specific complications (increased risk or history of nephrolithiasis) [28–30]. [31] With any restrictive diet, the potential micronutrient deficiencies need to be considered and supplementation is likely to be required [24]. Thus, a ketogenic dietary prescription in a clinical trial of ADPKD must take these factors into consideration [5]. Other authors have suggested that starting with a less restrictive ketogenic diet (e.g. a 1:1 or 2:1 ratio of fat: protein plus carbohydrates rather than the traditional 4:1 ratio) may enhance adherence and reduce complications [23].

In the following section specific dietary considerations and safety aspects for people with ADPKD are provided, with recommendations for nutritional targets and strategies to design an ADPKD-appropriate ketogenic diet intervention. This section is based on current standard of care for diet in ADPKD, data from published clinical trials of ketogenic diet in ADPKD, and expected co-morbidities in ADPKD (summarized in Table 4).

**Table 4:** Summary of recommendations for developing a ketogenic diet in ADPKD

Dietary constituent	Recommendation	Justification
Macronutrients		
Carbohydrate	< 50 g available carbohydrate per day	Achieve ketosi s
Protein	Type: Inclusion of plant-based proteins, with concomitant reduction in animal-based proteins Amount: < 1 g/kg/day total protein	May assist to slow CKD progression, particularly in those with eGFR < 30 ml/min/1.73m^2^ Reduce risk of nephrolithiasis Reduce phosphorus intake
Dietary fat	Type: Reduce saturated fats and trans fats and replace with polyunsaturated fats and monounsaturated fats through increased plant-based eating and regular inclusion of fish Amount: Limit saturated fat and trans fats to < 10% of total energy intake	CVD-risk reduction
Micronutrients		
Sodium	Type: Limit high salt food and foods with added salt Amount: < 2300 mg per day	Slowing CKD progression through blood pressure lowering Reduce risk of nephrolithiasis CVD-risk reduction
Potassium	Type: Include whole foods from plant-based sources, such as nuts, seeds, low carbohydrate fruits and vegetables. Limit ultra-processed foods containing potassium additives Amount: Restriction only in the setting of hyperkalemia. In event of hyperkalemia the cause needs careful evaluation, limiting dietary intake only when required. Thorough dietary assessment is required to appropriately address potential dietary causes	Adequate potassium for CVD risk reduction Electrolyte disturbances in advanced CKD
Phosphorus	Type: Choose plant-based proteins that contain low bioavailable phosphorus. Limit ultra-processed foods that contain phosphate additives and reduce animal-based proteins Amount: Restriction in the setting of hyperphosphatemia	Electrolyte disturbances and CKD progression
Dietary fiber	Type: Increase plant-based foods, including nuts, seeds, low carbohydrate fruits and vegetables Amount: aim for 25-40 g total fiber per day, including 7-13 g soluble fiber per day. Rich sources of soluble fiber include low carb legumes, vegetables, fruits, and psyllium husk	Slowing CKD progression through reduction of metabolic acidosis CVD-risk reduction
Phytosterols	Type: Include plant-based foods containing natural phytosterols. Consider phytosterol-enriched foods if hypercholesterolemic Amount: 2 g per day	CVD-risk reduction
Fluid	Type: Fluid should be predominantly water Amount: Drink to thirst and aim to produce 2-3L of urine per day In eGFR < 30 ml/min/1.73 m^2^, an individualized approach to fluid management is necessary	Fluid balance Vasopressin suppression Reduce risk of nephrolithiasis

### Dietary protein

*Standard Guidelines for Protein intake in ADPKD:* Protein metabolism and clearance is disrupted in kidney disease, with experimental models showing that long-term dietary protein intake > 1.5 g/kg/day resulted in glomerular hyperfiltration, inflammation, and proteinuria, which are all known risk factors for progressive kidney function loss [32]. However, post hoc analyses of 200 people with ADPKD from the MDRD study reported no effect of protein intake on kidney function in people with eGFR 25-55 ml/min/1.73 m^2^, and only a marginal effect in those with eGFR 13-24 ml/min/1.73 m^2^.[6] These data have informed the recommendations for moderate protein consumption (0.75-1 g/kg/day) for people with ADPKD [5, 10].() Due to insufficient evidence, there is no recommendation regarding the type of protein, [32] although there is increasing interest and research evaluating the impact of plant-based diets due to the potential benefits on metabolic acidosis and progression of kidney disease [33, 34].

*Protein content of ketogenic diets:* Most traditional ketogenic diets derive between 10 to 35% of total energy intake from protein [23], with sources recommending at least 1.0–1.5 g/kg/day protein to preserve lean body mass [35]. There are no recommendations regarding the sources of protein (i.e., animal- or plant-based) in mainstream ketogenic diets, although plant-based ketogenic diets have been highlighted for ADPKD [36].

*Recommendations for dietary protein in ketogenic diet in an ADPKD clinical trial:* Given the potential harm of high protein content of traditional ketogenic diet[23, 35], when designing a ketogenic diet for ADPKD, we suggest a modest intake of 0.75-1 g protein/kg/day (in line with current standard of care) with inclusion of plant-based proteins [5, 36].

### Dietary sodium

*Standard Sodium intake in ADPKD.* Dietary restriction of sodium intake is a cornerstone of management in ADPKD due to its effects on hypertension and reduction in kidney cyst growth [37]. Increased salt intake hastens disease progression through stimulating an increase in vasopressin[38] and pressure [28, 37, 39]. The HALT-PKD study, [37] a RCT evaluating the impact of intensive blood pressure control, recommended dietary salt restriction for all participants, irrespective of treatment arm. Post hoc analyses showed that higher urinary sodium excretion was associated with increased total kidney volume (in participants with eGFR > 60 ml/min/1.73 m^2^), and greater kidney function decline and progression to renal composite end-point for those with moderate kidney dysfunction (eGFR 25-60 ml/min/1.73 m^2^) [37]. Current dietary guidelines recommend a sodium restriction of < 2300 mg per day for people with hypertension, CKD and ADPKD [5, 10, 13, 32, 37, 40, 41].

*Sodium content of ketogenic diets*. There is no predefined guideline for sodium content of ketogenic diets. However, readily available ketogenic diet-related websites frequently recommend that people increase their sodium intake to 2000-5000 mg daily. An international consensus of ketogenic diet experts, including doctors and dietitians, recommended that sodium only be added to foods if patients become hypotensive [42].

*Recommendations for dietary sodium in ketogenic diet in an ADPKD clinical trial:* Given the lack of evidence, we suggest that dietary sodium intake should be maintained at < 2300 mg per day, as per ADPKD guidelines [10]. Of note, consuming less than 1500 mg is not recommended, however clinical trials are needed to determine the optimal level and impact of sodium in an ADPKD-appropriate ketogenic diet. Dietary counseling should include meal plans and recommendations that minimize the use of high salt-content foods and limit added salt. We also recommend that a protocol be embedded within a clinical trial that allows the addition of dietary sodium above 2300 mg daily for a defined period should a participant develop orthostatic hypotension. Keto-flu symptoms should first be treated with increased electrolytes such as magnesium and potassium, and adequate fluid intake.

### Dietary potassium

*Standard Guidelines for Potassium intake in ADPKD.* During the early stages of ADPKD, increased dietary potassium of up to 4000 mg per day could benefit blood pressure management, reduce cardiovascular disease (CVD) risk[18], and minimize metabolic acidosis [33]. Unless hyperkalemia is present, restriction of dietary potassium intake is not required [32].

*Potassium content of ketogenic diets*. The KETO-ADPKD study prescribed an upper limit of 4000 mg potassium daily [27]. General websites promoting ketogenic diets recommend consuming 3000-4000 mg daily, in line with dietary guideline recommendations.

*Recommendations for potassium in a ketogenic diet in an ADPKD clinical trial:* In the early stages of ADPKD (eGFR > 30 ml/min/1.73 m^2^) there is no indication to reduce potassium intake. Should a ketogenic diet be trialed in more advanced ADPKD (eGFR < 30 ml/min/1.73 m^2^), regular monitoring for hyperkalemia should be maintained with appropriate dietary strategies implemented when indicated.

### Dietary phosphorus

*Standard Guidelines for Phosphate intake in ADPKD.* Phosphorus homeostasis is dysregulated early in CKD resulting in a positive phosphorus balance and vascular calcification.[43] In experimental models of ADPKD, lower dietary phosphorus intake slows cystogenesis and inhibits the activation of fibrotic pathways [44]. An observational study (DIPAK) of 604 people with ADPKD reported kidney phosphorus wasting being prevalent in 59% of participants, associated with disease progression (mean difference of −0.7 ml/min/1.73 m^2^ per year); they theorized kidney phosphorus wasting to be a marker of early proximal tubule dysfunction [45]. However, interpretation of this study with respect to dietary recommendations is difficult as phosphorus wasting was calculated using urinary phosphorus excretion, which is affected by dietary phosphorus intake that was not measured. In line with general CKD dietary guidelines[32], for people with ADPKD it is recommended to limit both dietary sources of phosphorus additives and intake of animal-based phosphorus (e.g., meat, poultry, dairy) due to the high bioavailability of these sources of phosphorus. (Chebib 2018) Observational studies suggest that plant-based diets may assist with maintaining phosphate balance due to the lower bioavailability of phosphorus [32].

*Phosphate content of ketogenic diets*. Phosphorus content of ketogenic diets is rarely reported in ketogenic diet trials. The KETO-ADPKD study prescribed an upper limit intake of 700 mg daily[27], less than the 1000 mg daily recommended by general dietary guidelines.

*Recommendations for phosphate in a ketogenic diet in an ADPKD clinical trial.* A focus on a reduction in foods containing phosphorus-additives and the inclusion of lower bioavailable phosphorus sources (e.g., plant-based foods) is recommended to maintain phosphate balance. Regular monitoring of serum phosphorus levels should be continued.

### Fluid intake

*Standard Guidelines for fluid intake in ADPKD*. The largest RCT (PREVENT-ADPKD) reported that increasing fluid intake (2-3L/day) alone was safe but not effective in slowing disease progression in ADPKD. (Rangan 2022). However, based on other factors (safety, patient risk for nephrolithiasis and values and preferences of consumers/clinical experts), the current ADPKD KDIGO guidelines suggest maintaining adequate fluid intake (2-3L/day) in patients with eGFR > 30 mL/min/1.73 m^2^.[10] Whilst increasing fluid intake was thought to reduce vasopressin levels, the largest RCT (PREVENT-ADPKD) reported no change in serum copeptin levels, a surrogate measure of vasopressin, despite significant reductions in urine osmolarity and increase in urine volume. (Rangan 2022) In advanced stages of CKD (< 30 mL/min/1.73 m^2^) a personalized approach to fluid intake was suggested [46].

*Water intake in ketogenic diets*. In non-ADPKD populations it has been recommended that participants following a ketogenic diet consume at least 2L of fluid daily to reduce the risk of nephrolithiasis [47], whilst the KETO-ADPKD study[27] prescribed 3L of fluid daily for participants in all arms of the study, including the ketogenic diet arm.

*Recommendations for water intake in a ketogenic diet in an ADPKD clinical trial.* Recommendations should be based on the KDIGO guidelines to ensure adequate fluid intake [10]. Water intake should be monitored in the trial groups to ensure that there are no differences.

### Micronutrients

*Standard Guidelines for micronutrients in ADPKD.* There are no ADPKD-specific micronutrient guidelines. Nutrition guidelines for CKD do not recommend routine supplementation of micronutrients, due to potential toxicity or lack of clinical efficacy, unless a person is deficient [32].

*Micronutrients in ketogenic diets.* Compared to control diets, trials evaluating carbohydrate-restricted diets have shown lower intakes of thiamine, folate, calcium, magnesium, iron and iodine, with the intake of these micronutrients being below recommended daily intake targets [48].

*Recommendations for micronutrient intake in a ketogenic diet in an ADPKD clinical trial.* Whether an ADPKD-appropriate ketogenic diet can be adequate in micronutrients needs evaluation. Regular monitoring of micronutrient intake and status should be undertaken throughout a clinical trial to evaluate adequacy.

## Safety considerations of ketogenic diets in ADPKD

As discussed earlier, increased rates of adverse events, such as dyslipidemia and nephrolithiasis, are concerns to implementing a ketogenic diet in ADPKD. A ketogenic meal plan needs to incorporate dietary strategies to mitigate these risks, with monitoring and evaluation as required in a clinical trial.

### Dyslipidemia

Ketogenic diets are inherently high in fat, comprising 60–90% of total energy. People with ADPKD commonly have hyperlipidemia, increasing their risk of CVD and CVD-mortality [29, 49]. Clinical trials have shown elevations of LDL-C between 6–35% ^37–41^ following the use of ketogenic diets, although this effect is not consistent [50]. In the KETO-ADPKD trial, 17% of participants receiving a ketogenic diet developed hypercholesterolemia and 12% developed hypertriglyceridemia [27]. Thus, an ADPKD-appropriate ketogenic diet needs to be designed utilizing guidelines for managing dyslipidemia, namely modifying the type and amount of fat consumed, increasing soluble dietary fiber intake, and incorporating dietary phytosterols [51, 52].

Reductions in saturated fat intake (e.g., fats found in meat, dairy and many packaged foods) and a subsequent increase in polyunsaturated fatty acids (e.g., fats found in nuts, seeds, wholegrains and fatty fish) have been shown to reduce LDL-C [52]. In addition, it is recommended that people limit their total fat intake to less than 40% of total energy and saturated fat to less than 10% of total energy, or less than 7% of total energy if hypercholesterolemia is present [52]. The focus on polyunsaturated fatty acid dietary sources is particularly important, given that ketogenic diets require > 60% of total caloric intake from dietary fat sources.

Soluble fiber can lower total and LDL-cholesterol, and slow the absorption of carbohydrates and fats [52]. Ensuring a dietary fiber intake of at least 25 g per day, and particularly 7-13 g of soluble fiber, is associated with cholesterol-lowering and heart health benefits [51].

Phytosterols are compounds similar in structure and function to cholesterol and naturally occur in plant-based foods, including vegetable oils, nuts, and seeds. Meta-analyses demonstrated that an intake of 600 mg to 2000 mg of phytosterols daily can reduce LDL-C by 6–12% in a dose-dependent manner [53]. These higher doses can only be achieved through the consumption of both natural and sterol-enriched food sources.

*Recommendation for a ketogenic diet to mitigate dyslipidemia in an ADPKD Clinical Trial*: To minimize the negative consequences of the required high fat intake, an ADPKD-appropriate ketogenic diet should limit the consumption of animal-based foods to minimize saturated fatty acid intake to < 7% of energy, and should include monounsaturated fatty acid- and polyunsaturated fatty acid-rich plant-based foods and fish as the primary source of dietary fat. In addition, very low carbohydrate, high fiber fruits, vegetables, nuts, and seeds will be needed to achieve an adequate fiber intake of > 25 g daily. Low carbohydrate, phytosterol-enriched foods or supplements may help to achieve cholesterol-lowering effects. Lipid levels should be monitored regularly while following a ketogenic diet. Extreme hypercholesterolemia at baseline may be a contraindication to commencing a ketogenic diet, and persistent dyslipidemia may be an indication to cease a ketogenic diet.

### Nephrolithiasis

The risk of kidney stones is increased 5–10-fold in ADPKD due to abnormal tubular structure and metabolic risk factors (hypocitraturia, hyperoxaluria, hyperuricosuria and low urine pH) [30, 54]. The most common stones in ADPKD, i.e., uric acid and calcium oxalate [55], are positively associated with increasing total kidney volume[54]. Recurrent stones are also more common as kidney function declines [56]. Nephrolithiasis increases morbidity due to flank pain, hematuria and urinary tract infections[30] and hastens decline in kidney function [57].

A systematic review and meta-analysis of 36 studies involving 2795 healthy participants on ketogenic diets demonstrated an estimated pooled incidence of kidney stones of 5.9% (95% CI 4.6–7.6%, I^2^ = 47%) over a mean follow up time of 3.7 ± 2.9 years; compared to an annual incidence of nephrolithiasis of 0.25–0.3% in the general population [25]. Similar to stone formation in the ADPKD population, the most common type of kidney stones following a ketogenic diet included uric acid (48.7%), calcium-based (36.5%), and mixed uric acid and calcium-based stones (27.8%) [25]. The mechanism of nephrolithiasis following a ketogenic diet is unclear, but likely related to hypocitraturia, low urine pH, and acidosis due to consuming a high protein and low-alkali diet [24, 25].

Providing patients with general advice to ensure adequate fluid intake to produce 2L of urine daily is recommended to reduce the recurrence of kidney stones, regardless of type [58]. High dietary salt intake induces a rise in urinary calcium and reduces urinary excretion of citrate, with high protein intake having an additive effect [59]. Total protein should be limited to 0.8 g/kg/day, with a preference for plant-based proteins and a reduction in animal-based proteins due to their beneficial lithogenic profile [60, 61]. Other general dietary recommendations to reduce the risk of nephrolithiasis include increased fruit and vegetable intake, and maintaining moderate calcium intake (1.2 g/day) [62].

Calcium-oxalate stones require adequate dietary calcium to bind oxalate in the intestinal lumen [61]. Moderate to high oxalate-containing foods should be consumed in combination with adequate calcium intake to reduce bioavailability and absorption, and very high oxalate-containing foods should be avoided. Supplementation with citrates, such as potassium citrate, has also been reported to reduce the rate of recurrent calcium-containing stones (RR 0.26, 95% CI 0.10–0.68; 7 studies, n = 477) [63]. The use of citrate has also been shown to slow cyst growth and ADPKD progression in juvenile rats [64].

*Recommendation for a ketogenic diet to mitigate nephrolithiasis in an ADPKD clinical trial*: Therefore, we suggest that an ADPKD-appropriate diet should be rich in plant-based foods while limiting total protein intake to 0.75–1.0 g/kg/day, reduced in salt (< 2300 mg daily), and adequate in calcium. For people with a history of calcium-oxalate stones, a reduction in dietary oxalates and use of a citrate-based supplement may be required. Given the deleterious impact of nephrolithiasis on kidney function, recurrent stones may be a contraindication to trialing a ketogenic diet.

### Hypertension management

Hypertension is common and manifests prior to a loss in kidney function in > 60% of adults with ADPKD [28]. Salt restriction is the primary dietary strategy used to control blood pressure in patients with CKD. In a CKD population, increased fruit and vegetable intake improved systolic blood pressure in general CKD, with pooled analyses indicating an average − 5.6 mmHg (95% CI, − 8.3 mmHg to − 2.8 mmHg) [32]; however this has not been specifically tested in people with ADPKD, and only 3 studies involving people with CKD have been identified [32]. Whilst a meta-analysis of 23 RCTs in the general population did not show significant changes to blood pressure following a ketogenic diet [65], the only clinical trial in ADPKD found that 17% of participants reported transient orthostatic symptoms after commencing the diet [27].

*Recommendation for a ketogenic diet to mitigate hypertension in an ADPKD Clinical Trial*: Given the increased risk for hypertension, a ketogenic diet that incorporates moderate sodium intake (< 2300 mg daily) with adequate very low carbohydrate fruit and vegetables is recommended. Blood pressure and anti-hypertensive medications should be reviewed regularly and adjusted if required to minimize orthostatic symptoms.

## Considerations for clinical trial design

### Inclusion and exclusion criteria

When selecting appropriate participants to trial a ketogenic diet there are several clinical presentations that should exclude participation. The following list is not exhaustive, but outlines clinical presentations most relevant to people with ADPKD:

- Recurrent nephrolithiasis [42].
- Baseline refractory dyslipidemia, particularly if the person has a low body weight [42, 66]. Extra caution is recommended when implementing a ketogenic diet that is not for weight loss purposes in people who do not have excess weight and who present with baseline metabolic abnormalities [66].
- Malnutrition or low body weight. Malnutrition is associated with loss of kidney function of > 3 ml/min/1.73 m^2^/year [67], and the rate of malnutrition increases as kidney function declines [68]. In a cohort of 288 people with ADPKD (average eGFR of 65 ml/min/1.73 m^2^) approximately 30% were identified as being at risk of malnutrition or were already malnourished, and those who had a height adjusted TKV (HtTKV) > 2340 ml/m had an 8.7 times greater risk of being malnourished, independent of kidney function [68].
- Pregnancy or breastfeeding [66].
- Type 1 diabetes due to the increased risk of diabetic ketoacidosis [66].

### Trial duration

Trials with sufficient duration are required to assess the safety and effectiveness of a ketogenic diet in ADPKD on surrogate endpoints (eGFR, Ht-TKV). Change in kidney function is a core outcome measure in kidney disease trials [69]. Increase in TKV is a widely accepted marker of ADPKD progression, with meaningful changes being detected after 12 months [70]. Therefore, a trial should be at least 12 months long. Whether adequate adherence to a complex diet can be maintained for this length of time needs to be evaluated.

### Safety monitoring

Given these complex clinical considerations, participants with ADPKD commenced on a ketogenic diet in a clinical trial should undergo biochemical monitoring (full blood count, electrolytes, kidney and liver function tests, and fasting lipid profiles) before, and regularly throughout the intervention, particularly in the first 12 months [42]. For people with moderate-to-advanced CKD (eGFR < 30 ml/min/1.73 m^2^), regular monitoring for electrolyte disturbances is recommended due to the lack of safety data for this population. Regular self-monitoring of ketone bodies, through either breath or finger-prick, should be encouraged to ensure participants are achieving ketosis.

### Psychosocial impacts

Consumer involvement and co-design should be embedded from development to evaluation of a ketogenic diet trial to ensure safety, feasibility and acceptability. The acceptability and potential burden of a ketogenic diet should not be ignored and needs further evaluation in qualitative studies. People with ADPKD experience physical symptoms and emotional distress related to their diagnosis, progression, and treatment options [4], therefore it is necessary to document the participant experience and patient-reported outcomes.

### Adherence

Barriers to adhering to the ketogenic diet include difficulties eating out and eating in social situations, leading to approximately 40% of participants taking breaks from the ketogenic diet [36, 71]. “Keto-flu” appears to be transient and generally not a barrier to continuing the ketogenic diet [27]. Regular monitoring and support from experienced dietitians throughout a trial are needed to support adherence [71]. Self-monitoring of ketones may also provide ongoing feedback to participants. Additionally, the cost and accessibility of the necessary food items to follow a ketogenic diet have not been described. Future trials should include more detailed evaluation of participant experiences and long-term adherence to the diet.

## Practical application for clinical trial design

Given the complex clinical and nutritional needs of people living with ADPKD, careful design of a ketogenic diet in a clinical trial is required. The nutrient recommendations contained herein are based on synthesis of dietary recommendations for CKD and common co-morbidities, including hypertension, dyslipidemia and nephrolithiasis. Specific nutrient recommendations to guide the development of an ADPKD-appropriate ketogenic diet for future clinical trials in ADPKD are outlined in Table 4. A sample one-day meal plan of a PKD-appropriate ketogenic diet, based on these nutrient recommendations, is provided in supplementary file 1.

## Conclusion

This review comprehensively provides guidance on developing an ADPKD-appropriate ketogenic diet. The balance of potential benefits to risks of the ketogenic diet must be considered prior to conducting a clinical trial. This review has highlighted several important nutritional considerations that need to be taken into account when designing a ketogenic diet in clinical trials in ADPKD. Further evaluation of whether such a nuanced ketogenic diet can be developed and be nutritionally adequate, as well as consumer’s perspectives regarding the design of a trial is required.

## Supplementary Information

Below is the link to the electronic supplementary material.

Supplementary file1 (DOCX 17 KB)

## Data Availability

N/A.
