# Supplementary material for: Nutritional considerations for designing ketogenic dietary interventions for people with Autosomal Dominant Polycystic Kidney Disease
Source: J Nephrol. 2025 Aug 10;38(8):2173–87. doi: 10.1007/s40620-025-02378-3 (PMC12630169; doi:10.1007/s40620-025-02378-3)
Supplement: Supplementary file 1 — Supplementary file1 (DOCX 17 KB) [file 40620_2025_2378_MOESM1_ESM.docx]

| **Supplementary File 1:** Sample 1-day meal plan for a 65 kg female following a PKD-Keto diet. | | |
| --- | --- | --- |
| **Meal** | **Recipe** | **Ingredients** |
| **Breakfast** | Sauteed mushrooms on keto bread | 1 serve of keto bread  1 cup mushrooms  1 shallot  2 tsp olive oil  2 tsp margarine  0.25 tsp salt |
| **Snack 1** | Raspberry Smoothie | 0.5cup raspberries  0.5c skim milk  2 tbls reduced fat Greek yoghurt |
| **Lunch** | Nutty Green Salad | 2cup lettuce  0.25c peas  0.25c onion  0.25c snow peas  0.5 avocado  1 tabls mayonnaise  1 tbls olive oil  2 tsp pickles  2 tsp lemon juice |
| **Snack 2** | Cucumber with pesto | 1 cup cucumber  2 tbls basil pesto |
| **Dinner** | Tuscan Barramundi | 100g cooked barramundi  0.25c kale  0.25c onion  1 tbls sundried tomato  0.5c skim milk  3 tsp olive oil |
| Meal plans were constructed for 7 days. For a 65kg female meal plans provided an average daily intake of 1900kcal, 66g protein, 155 g total fat, 50g carbohydrate, 37g fiber, 2030mg sodium, 89mmol potassium and 1200mg phosphorus.  *Menu needs to be adapted according to local food habits, food preferences and social-economic conditions | | |
